# Complete genome sequence of a *Neisseria meningitidis* isolate from an ankle joint with septic arthritis

**DOI:** 10.1128/mra.00111-25

**Published:** 2025-04-29

**Authors:** Eduardo Rubio-Mora, María Amoroto-Bengoetxea, Alicia Rico Nieto, Alejandro Díez-Vidal, Jesús Mingorance, Inmaculada Quiles-Melero

**Affiliations:** 1 Servicio de Microbiología y Parasitología. Hospital Universitario La Paz 16268, Madrid, Spain; 2 Instituto de Investigación Hospital Universitario La Paz (IdiPAZ) 638528 , Madrid, Spain; 3 Unidad de Enfermedades Infecciosas, Hospital Universitario La Paz 16268, Madrid, Spain; 4 CIBERINFEC, Instituto de Salud Carlos III 38176, Madrid, Spain; Rochester Institute of Technology, Rochester, New York, USA

**Keywords:** *Neisseria meningitidis*, septic arthritis, hybrid assembly

## Abstract

***N****eisseria meningitidis* has been identified as the causative agent of a wide variety of infections. We report the complete genome sequence of an *N. meningitidis* isolate from a joint fluid sample collected from a patient with primary meningococcal septic arthritis. This strain belongs to the sequence type 17488 of the ST-11 clonal complex, and its assembled genome consists of 2,242,622 base pairs with 51.75% GC content.

## ANNOUNCEMENT

*Neisseria meningitidis* can colonize the nasopharynx in humans and has been identified as the causative agent of a wide variety of infections, from meningitis and meningococcemia to endocarditis, pneumonia, and septic arthritis (1). Different manifestations of meningococcal septic arthritis (MSA) have been described (2). Primary MSA, emerging without systemic symptoms, is rare and represents a critical orthopaedic emergency necessitating immediate surgical joint drainage alongside antimicrobial therapy (3). This study presents the complete genome sequence of an *N. meningitidis* isolate from an ankle joint of a patient with primary MSA.

*Neisseria meningitidis* was isolated from synovial fluid after 48 hours of aerobic growth on blood agar. Identification was confirmed by MALDI-TOF. Gradient strip testing (bioMérieux, Marcy-l'Étoile, France) with CLSI (4) and (5) breakpoints showed susceptibility to all tested antibiotics: penicillin (MIC <0.03), ampicillin (MIC <0.06), cefotaxime (MIC <0.01), meropenem (MIC <0.25), ciprofloxacin (MIC = 0.004), and rifampicin (MIC <0.01).

Genomic DNA extraction was performed using the DNeasy Blood & Tissue Kit (Qiagen, Hilden, Germany) following the manufacturer’s instructions. DNA was sequenced with MinION (Oxford Nanopore, Oxford, UK) and Ion GeneStudio S5 (Ion Torrent, Thermo Fisher Scientific) platforms.

For Nanopore sequencing, DNA was end-repaired using the NEBNext companion module (New England Biolabs, USA), followed by barcode and adapter ligation with the Native Barcoding Kit 24 v14 (Oxford Nanopore Technologies, UK). Sequencing was done on a MinION Mk1C using a Flongle flowcell (Oxford Nanopore Technologies, UK) and MinKNOW v23.07.12 and Guppy v7.1.4 software.

For Ion Torrent sequencing, DNA was fragmented with an M220 Focused Ultrasonicator (Covaris, MA, United States). End-repair, barcoding, and adapter ligation were done using the NEBNext Fast DNA Library Prep Set (New England Biolabs, USA). Fragment size selection was done using E-Gel Electrophoresis System (Thermo Fisher Scientific, MA, United States) to recover fragments of 350 base pairs. Short-read sequencing was done on an Ion GeneStudio S5 using an Ion530 Chip.

Hybrid genome assembly was done with short and long reads using Unicycler v0.5.0 (4). Assembly was assessed using QUAST v5.0.2 (6). Kraken2 v2.1.2 (7) was used to assess contamination. Annotation was done using Prokka v1.14.6 (8). Abricate v1.0.1 (9) was utilized to search for antimicrobial resistance genes and PubMLST for typing (10). Default parameters were used in all tools.

QUAST analysis revealed 22 contigs with a total length of 2,242,622 base pairs, with 51.75% GC content, an L50 = 1 and an N50 = 2.002.742, and 101.5 mean coverage. Kraken2 confirmed that the sequences were free of contamination. Prokka identified 2,230 coding sequences, 6 rRNA operons, and 60 tRNA genes. No antimicrobial resistance genes or mutations were detected, in agreement with the results of antibiotic susceptibility testing. Searches done in the PubMLST database for *Neisseria* typing identified the sequence type ST17488, which belongs to the ST-11 clonal complex, the capsule group W, and the Bexsero Antigen Sequence Typing profile 3543.

## Data Availability

The raw short-read and long-read sequences that support the findings of this study are openly available in the Sequence Read Archive (SRA) database, hosted by the National Center for Biotechnology Information, under the BioProject accession number PRJNA1085389.
